# The Southern Medical College

**Published:** 1885-09-20

**Authors:** 


### THE SOUTHERN MEDICAL COLLEGE.

The Seventh Annual Session of this excellent school will commence on the 6th of October, proximo. The opening address will be delivered by Prof. A. G. Hobbs. in the College Auditorium, at 11 o'clock a. m. We are informed that there is a prospect of a large class the present term.

